# A Large Cross-Sectional Community-Based Study of Newborn Care Practices in Southern Tanzania

**DOI:** 10.1371/journal.pone.0015593

**Published:** 2010-12-21

**Authors:** Suzanne Penfold, Zelee Hill, Mwifadhi Mrisho, Fatuma Manzi, Marcel Tanner, Hassan Mshinda, David Schellenberg, Joanna R. M. Armstrong Schellenberg

**Affiliations:** 1 Faculty of Infectious and Tropical Diseases, London School of Hygiene and Tropical Medicine, London, United Kingdom; 2 Institute of Child Health, University College London, London, United Kingdom; 3 Ifakara Health Institute, Dar es Salaam, Tanzania; 4 Swiss Tropical and Public Health Institute, Basel, Switzerland and University of Basel, Basel, Switzerland; 5 COSTECH, Dar es Salaam, Tanzania; Kenya Medical Research Institute/Wellcome Trust Research Programme, Kenya

## Abstract

Despite recent improvements in child survival in sub-Saharan Africa, neonatal mortality rates remain largely unchanged. This study aimed to determine the frequency of delivery and newborn-care practices in southern Tanzania, where neonatal mortality is higher than the national average. All households in five districts of Southern Tanzania were approached to participate. Of 213,220 female residents aged 13–49 years, 92% participated. Cross-sectional, retrospective data on childbirth and newborn care practices were collected from 22,243 female respondents who had delivered a live baby in the preceding year. Health facility deliveries accounted for 41% of births, with nearly all non-facility deliveries occurring at home (57% of deliveries). Skilled attendants assisted 40% of births. Over half of women reported drying the baby and over a third reported wrapping the baby within 5 minutes of delivery. The majority of mothers delivering at home reported that they had made preparations for delivery, including buying soap (84%) and preparing a cloth for drying the child (85%). Although 95% of these women reported that the cord was cut with a clean razor blade, only half reported that it was tied with a clean thread. Furthermore, out of all respondents 10% reported that their baby was dipped in cold water immediately after delivery, around two-thirds reported bathing their babies within 6 hours of delivery, and 28% reported putting something on the cord to help it dry. Skin-to-skin contact between mother and baby after delivery was rarely practiced. Although 83% of women breastfed within 24 hours of delivery, only 18% did so within an hour. Fewer than half of women exclusively breastfed in the three days after delivery. The findings suggest a need to promote and facilitate health facility deliveries, hygienic delivery practices for home births, delayed bathing and immediate and exclusive breastfeeding in Southern Tanzania to improve newborn health.

## Introduction

Despite recent dramatic improvements in child survival in sub-Saharan Africa, neonatal mortality rates (death within the first month of life) remain largely unchanged [1]. Ninety-nine percent of all newborn deaths occur in low- and middle- income countries, with two-thirds of those occurring in Asia and Africa [2]. Improvements in neonatal mortality rates are essential if countries are to meet their targets for Millennium Development Goal 4 [2]. The main causes of neonatal mortality are intrapartum-related deaths, complications of pre-term birth or low birth weight, sepsis and pneumonia (estimated proportions of 23%, 29%, 15% and 10% respectively) [1]. Around three-quarters of neonatal deaths are in the first week of life [2].

The World Health Organization recommended essential newborn care behaviours include hygienic practices at delivery (clean hands and delivery surface, nothing unclean to be introduced into the vagina) and for the umbilical cord (clean cutting and tying instruments and applying nothing to the cord), thermal care (immediate drying and wrapping of the baby after delivery, skin-to-skin contact with the mother), extra care for low birth-weight/preterm birth (additional warmth, cleanliness and nutrition and early recognition of diseases) and early and exclusive breastfeeding to reduce the risk of the main causes of neonatal deaths in both community and facility deliveries [3]. The frequency of reported practice of these behaviours varies widely between countries. Osrin et al (2002) measured the frequency of several newborn care behaviours as part of a trial of a community-based participatory intervention in Nepal to improve newborn care. Around half of respondents reported that the birth attendants washed their hands before the birth of the baby, 64% reported that their newborns were wrapped within half an hour of birth and 92% had been washed within an hour of birth. Breastfeeding rates within an hour of birth were high (91%) [4]. Frequencies of newborn care behaviours were also assessed before the implementation of a community-based maternal and newborn health intervention in rural northern India in 2003 [5]. Around one third of respondents (women who had had a live birth in 2001–2002) practised clean cord care, while less than 4% reported to have practiced thermal newborn care and less than 4% reported that they initiated breastfeeding within an hour of birth (intervention and control areas combined). Data from different countries within Africa also varied. Results from studies in Ghana showed that for home deliveries hand washing before delivery (79%), cord cutting with a new razor blade (98%) and tying the cord with a new thread (90%) were commonly practised, whereas dry cord care (8%, home and facility deliveries), immediate drying (33%) and wrapping (24%) and delayed bathing were not (7%) [6], [7]. In a Ugandan study, where the majority of deliveries were not in facilities, only around half (57%) of women reported that the cord was cut with a new razor blade, whereas 99% reported that the cord was tied with clean material. Around half of respondents reported breastfeeding immediately after delivery (51%) and practicing clean cord care (51%) [8]. The evidence of reduced neonatal mortality risk for specific newborn care practices is greater for some, such as breastfeeding practices [9], extra care for small babies, for example ‘Kangaroo mother care’ [10], and delivery attendant hand washing [11], than others, such as thermal care and other infection prevention measures.

Although mortality rates in children younger than five years in Tanzania have decreased from 128 per 1000 live births in 2000 to 83 in 2004 [12], annually at least 51000 Tanzanian newborns die, and the national neonatal mortality has remained at between 32 and 40 per thousand live births since 1990 [13]. Nationally, Tanzanian demographic and health surveys (DHS) found that although more than 90% of women received some antenatal care (ANC), only around half of deliveries took place in a health facility and 59% of babies were breastfed immediately after delivery [14]. The neonatal mortality rate was estimated to be 43.2 per thousand live births in Tanzania (2001–4) [15]. In this area, although the vast majority of women reported to have attended ANC, nearly two-thirds (61%) of deliveries in the area did not take place in a health facility [15]. Qualitative research in the same area included reports of potentially harmful newborn behaviours such as dipping newborn babies in cold water to make them cry, putting substances on the cord to help it dry, and giving the baby other food before initiating breastfeeding as the colostrums was perceived as dirty or there was insufficient milk [16].

The aim of this study is to describe newborn care practices quantitatively in a 5-district, largely rural population of just under 1 million people in southern Tanzania. Understanding the frequency of delivery and immediate newborn care practices in the area is essential for informing the choice of target behaviours for interventions aiming to reduce neonatal mortality.

## Results

Of 213,220 women age 13–49 years we interviewed 196,330 (response rate 92% response rate), of whom 144,247 had ever given birth, 139,425 to a live baby. There were 22,243 women who had had a live birth in the year before the interview (15% of women who had ever given birth), and these respondents answered questions related to intrapartum and postpartum care for their most recent birth.

### Place of and skilled attendance at delivery

Health facility deliveries accounted for 9,046 (41%) of births and nearly all non-facility deliveries took place at home (12,624, 57% of deliveries, Table 1). When an attendant was present, she was most commonly a female relative or friend (8,681 births, 39% of deliveries). Skilled attendants (doctors, nurses, and/or midwives) altogether assisted 8,862 births (40% of deliveries). A small proportion of deliveries (758, 9%) did not take place in health facilities yet were reported to have been attended by a skilled attendant. Six hundred and thirty women (3% of deliveries) gave birth alone.

**Table 1:** Place of birth and attendance at delivery for women having a live birth in the past year.

	n	%
**Place of delivery**	**N = 22,243**	
Health facility	9,046	41
Hospital	6,475	29
Health centre	472	2
Dispensary	2,099	9
Other	13,197	59
At home	12,624	57
Another household	298	1
Another place	274	1
Don't know	1	0
**Birth assistant** *	**N = 22,240**	
Skilled attendant	8,862	40
Doctor	1,951	9
Midwife	3,059	14
Nurse	6,129	28
Other	13,378	60
Female relative or friend	8,681	39
Traditional birth attendant	6,824	31
No one	630	3

*More than one answer allowed.

### Birth preparedness

Over half of women (13,084, 59%) reported having planned where they would deliver their baby. The vast majority of those (11,940, 91%) said they planned to deliver at a health facility. Around half of those women who said they had planned where to deliver (7,376, 56%) delivered in the place they had planned.

We asked the 13,201 mothers who delivered at home if they had made any preparations for delivery (Table 2). Respondents frequently reported that they had made preparations for delivery including buying soap (11,087, 84%), preparing a new or washed cloth for drying the child (11,285, 85%) and cleaning the floor (9,633, 73%). Just over half of women who had delivered at home (7,630, 58%) said they had made plans in case of an emergency during delivery.

**Table 2:** Preparations made for delivery for deliveries taking place outside of health facilities.

Preparation step	N	%
	(N = 13,201)	
Soap	11,087	84
Cloth (new or washed) for drying child	11,285	85
Cloth or Mat (new or washed) at the place of delivery	9,166	69
Clean floor	9,633	73
Cloth (new or washed) for covering child	11,627	88
Plan for emergency delivery	7,630	58

### Cleanliness and hygiene practices during childbirth

Additional question regarding hygiene practices during delivery were asked of women who delivered at home.

Over half (7,690, 58%) of women who delivered at home reported that the attendant wore gloves (Table 3). Six thousand and forty-six (46%) respondents reported that the birth attendant washed his/her hands before delivery *and* used soap. The vast majority of respondents reported that they cut the cord with a razor blade (12,327, 96%), with most of those reporting that it was a new blade (12,188, 95% of those using a razor blade). Around half of respondents (6,449, 49%) reported that the cord was tied with a new thread.

**Table 3:** Hygiene practices at delivery for non-facility deliveries.

Hygiene practice	n	%
Hand washing practices of birth attendant*	**(N = 13,192)**	
Washed hands before delivery and used soap	6,046	46
Wore gloves	7,690	58
	**(N = 12,873)**	
New or boiled razor blade used to cut the cord	12,188	95
	**(N = 12,503)**	
Cord tied with new or boiled thread	6,449	49

*More than one answer allowed.

When looking at all deliveries, 6,183 (28%) women reported putting something on the cord to help it dry, with the most common substances reported to be traditional medicine (8% of all deliveries) and oil (7% of all deliveries).

### Practices relating to thermal care of newborn

Ten percent (2,166) of women reported that their baby was dipped in cold water immediately after delivery to check that it was healthy (Table 4). Forty-two percent of women (9,228) reported that the baby was dried and 27% (6,046) reported that the baby was wrapped within 5 minutes of delivery. Over half of babies (13,190, 59%) were bathed within 6 hours of birth, three-quarters of them in warm water.

**Table 4:** Thermal care practices for newborn babies.

Thermal care practice	n (N = 22,234)	%
Baby dipped in cold water to check it is healthy	2,166	10
Time to drying of baby		
Less than five minutes	9,228	42
5 to 15 minutes	5,722	26
16 to 30 minutes	1,444	7
More than 30 minutes	932	4
Don't know	4,906	22
Time to wrapping of baby		
Less than five minutes	6,046	27
5 to 15 minutes	8,019	36
16 to 30 minutes	2,279	10
More than 30 minutes	916	4
Don't know	6,046	22
Time to bathing of baby		
Less than one hour	7,391	33
1 to 6 hours	5,799	27
More than six hours	6,569	30
Don't know	2,472	11
Temperature of water to Bath baby		
Cold	5,358	24
Warm	15,023	68
Don't know	1,850	8

In non-facility deliveries women were asked where the baby was put at different stages of the delivery process. Both between the baby being born and the placenta being delivered and after the cord being cut the majority of women reported that the baby was put on the bed (3,094, 76% and 11,252, 87% respectively), with less than 1% of respondents (32 and 112 respectively) reporting that the baby was put on her chest.

### Breastfeeding

**Figure 1:**
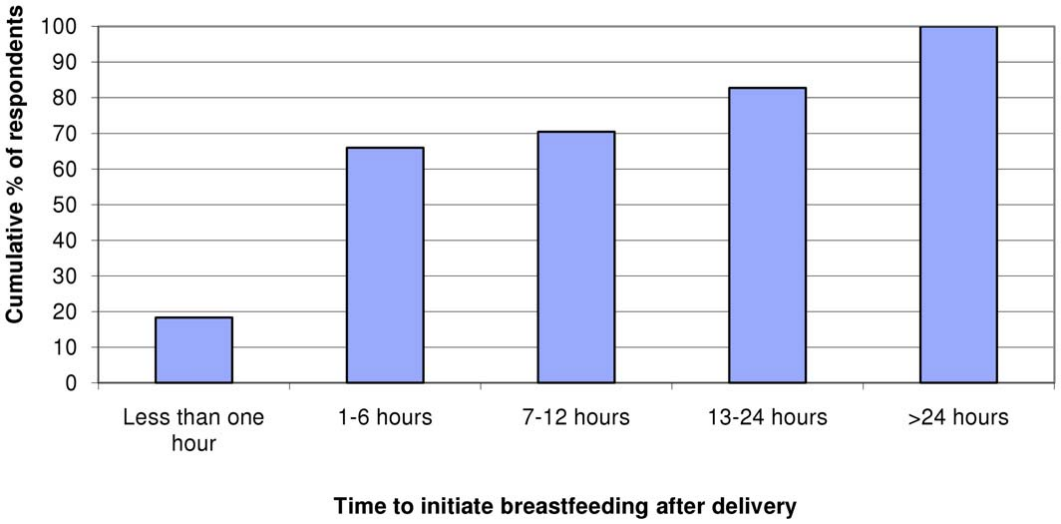
Time to initiate breastfeeding after delivery, cumulative proportion of respondents.

Eighty-three percent of women (18,330, 83%) reported initiating breastfeeding within 24 hours of delivery, although less than 20% (4,059, 18%) started to breastfeed within the recommended time period of an hour after delivery (Figure 1). Most commonly women reported breastfeeding between 1 and 6 hours of birth (10,556, 48%). Around half of women (11,247, 51%) reported giving their baby something other than breast milk in the first three days after delivery, most commonly sugar water (7,297, 65% of those giving other feeds).

## Discussion

Rates of both facility delivery and deliveries with a skilled attendant in this study are comparable to other findings from Tanzania [14]. Given that the majority of births occur at home and without a skilled attendant present it is encouraging that preparations such as buying soap and preparing cloths for the baby were common. Such preparations may explain the comparatively high levels of soap usage and immediate drying of babies after delivery. However, immediate wrapping was only practiced by around a third of respondents despite frequent cloth preparation, which demonstrates that preparation of items does not always lead to correct usage. The findings relating to preparations before delivery are in contrast to those from some Asian settings where women reported low levels of preparation. A lack of preparation was attributed to delivery preparations being embarrassing; pregnancy being considered a fragile state and there is the belief that the more people who know about an impending delivery the more delayed it will be [17].

One behaviour of concern in this study is the common act of bathing babies soon after birth. Furthermore ten percent of respondents reported dipping their baby in cold water to check it is healthy. The latter behaviour demonstrates a deliberate action despite warm water being readily available. Participants in qualitative research in Tanzania reported that they washed the baby immediately after delivery because they thought the baby was dirty, and that the use of cold water would make the baby cry or make the baby strong [18]. The reported perceptions that this behaviour is good for the baby, is encouraged by family members and is particularly important if there is an obvious vernix suggests newborn bathing behaviours could be difficult to change in this area [18].

The frequency of reported hygiene practices at delivery varied greatly. Women reported that fewer than half of birth attendants washed their hands using soap before delivery, whereas 96% of women reported that the umbilical cord was cut with a new or boiled blade. Over half of respondents reported that the umbilical cord was tied with a clean thread and over a quarter of respondents said they had put something on the cord to help it dry. These results demonstrate that some recommended hygiene behaviours are already widely practiced in Southern Tanzania, which may be a result of health education initiatives. For example, the common practice of using a clean blade to cut the cord may be through messages to prevent tetanus. Therefore newborn health interventions can afford to focus on other behaviours that are less well practiced in this area.

Our study also found fewer than 20% of respondents reported breastfeeding within one hour after delivery, although over half of babies had been breastfed by six hours after delivery and over 80% within 24 hours. These figures are much lower then those from the Tanzanian DHS findings in 2004, where 59% of babies were reported to be breastfed within one hour of birth and 92% within the first day [14]. The discrepancies between rates of breastfeeding within an hour of delivery may be partly due to differences between the three studies in the way the responses were coded. The DHS and this study both asked respondents to report time to initiate breastfeeding after delivery in hours. However, in addition to the duration of time, respondents for the DHS had the option of selecting a pre-coded answer ‘immediately’ when reporting the time to initiate breastfeeding. It could be argued that ‘immediately’ is an ambiguous time reference, meaning different respondents refer to different time periods when selecting this option [19]. Findings reported in this study suggest that many DHS respondents reporting that they ‘immediately’ breastfed may actually have initiated it a few hours after birth.

Qualitative data from Tanzania attributed delayed breastfeeding to a perception that colostrum was dirty and should not be given to the baby [16], or that there was a lack of milk immediately after delivery [18]. The practice of giving feeds other than breast milk to newborns immediately after delivery in southern Tanzania is of concern because of the increased risk of neonatal infections [9], [20], and the increased risk of HIV transmission [21], [22].

The strengths of this study include its large sample size and high response rate, giving a representative study sample. Such population-level findings for newborn care practices provide valuable knowledge for a health area where such data are currently scant, particularly in sub-Saharan Africa. Although the data are representative of the study area it is likely that frequencies of newborn care behaviours vary between different countries in sub-Saharan Africa, and within Tanzania. For example, findings from other parts of Tanzania suggest that bathing of newborns may be less common than found in this study [23]. Questions about delivery and newborn care practices were restricted to women who had delivered within a year preceding the interview to maximize recall accuracy. However, some reporting biases remained. For example, birth preparation steps are likely to have been over reported as women know they should have been making preparations (this is included in antenatal care in Tanzania). Also it is likely that the rates of hospital deliveries and deliveries attended by a skilled attendant have been over-reported due to misclassification. Anecdotal evidence suggests there is a tendency for dispensaries and health centres to be called hospitals, and facility staff members attending deliveries to be called nurse or midwife even if they are unskilled staff such as nurse assistants or other lay cadre. Also, attendants at home deliveries are sometimes referred to as midwives when they were in fact traditional birth attendants.

The questionnaire covered many delivery and care practices, with questions being administered in Swahili. A limitation of the tool includes the omission of questions on newborn care practices of low birth-weight babies. Also, the question used to investigate the frequency of applying items to the cord specifically asked about substances ‘to help the cord dry’. Although findings from formative research and qualitative data in the study area [16] found that this was a common reason, there may be other reasons for applying substances to the cord that would have been missed by the specificity of this question, and would mean the reported frequency of applying substances to the cord in this study is likely to have been lower than was actually the case.

This study highlights some of the difficulties in measuring newborn care practices. Firstly, it is unclear how accurately women recall time, especially immediately after delivery. This uncertainty is illustrated by the high proportion of women responding ‘don't know’ to questions about time in this study (e.g. 22% of respondents responded that they didn't know how long it was before their baby was dried or wrapped). Secondly, the issue of question constructs affecting responses is illustrated by the discrepancy between reported rates of breastfeeding in this study and DHS Tanzania, as described above. Furthermore, the questionnaire we used was translated and back translated a priori and administered in Swahili, whereas the DHS questionnaire was not, which may also have contributed to the different results seen. Such aspects raise concern over the validity of the reported frequencies, particularly of early breastfeeding, and drying and wrapping of the baby. Such difficulties were also reported by the Saving Newborn Lives technical working group when developing newborn health indicators [24].

Finally, the findings from this study present the frequencies of practiced behaviours, thus highlighting the areas with greatest potential for change. However, they do not explain why such behaviours are carried out and who are the people, circumstances or beliefs that influence the practice of them. Findings from Ghana [25], Tanzania [16], [18], [23] and India [26] show that these influences vary within and between counties. Therefore, in order to develop interventions to change newborn care behaviours it is necessary to conduct both quantitative and qualitative research in the planned implementation area.

In light of the above discussion our study has obtained important population-level information about delivery and newborn care practices in Southern Tanzania. Together with qualitative results from a similar area [16], [18], these findings provide valuable evidence to help develop and target community-based interventions to improve neonatal health in the area. In conclusion, the findings from this study suggest that promoting and facilitating health facility deliveries, hygienic delivery practices for attendants in home deliveries, delayed bathing and immediate and exclusive breastfeeding could be beneficial for newborn health in southern Tanzania. Further evidence is needed on the risk of neonatal death associated with sub-optimal newborn care practices.

## Materials and Methods

The study was undertaken within the framework of the assessment of the community effectiveness of Intermittent Preventative Treatment in Infants (IPTi), part of the IPTi Consortium (www.ipti-malaria.org, clinical trial number NCT00152204). We received ethical approval from local and national institutional review boards (Ifakara Health Institute and the National Tanzania Medical Research Co-coordinating Committee) through the Tanzania Commission for Science and Technology. Ethical and research clearance was also obtained from the London School of Hygiene and Tropical Medicine, UK, and the Ethics Commission of the Cantons of Basel-Stadt and Basel-Land, Switzerland. During field work, information sheets in Swahili about the study were given out, explaining why it was being done, by whom, and what it would involve. Consent to participate was obtained in writing from household heads and orally from women answering questions about their pregnancies. Confidentiality of all study participants was assured.

The study was conducted in the districts of Nachingwea, Lindi Rural, Ruangwa, Tandahimba and Newala Districts in Southern Tanzania, which had a total population of over 800,000 people in 2007. The study setting and field methods from a similar survey have been described in detail elsewhere [15], [16] so the key aspects are summarised here. The area has a wide mix of ethnic groups, including the Makonde, Mwera, Yao. Although most people speak the language of their own ethnic group, Swahili is also widely spoken. The most common occupations are subsistence farming, fishing and small scale trading. Cashew nuts, sesame and groundnuts are the major cash crops while food crops are cassava, maize, sorghum and rice. Most people live in mud-walled and thatched-roof houses; a few houses have corrugated iron roofs. Common water supplies are hand-dug wells which rely on seasonal rain, communal boreholes, natural springs and river water. Most rural roads are unpaved: some are not passable during rainy seasons while others are too steep for vehicles to pass. In 2000–2001 39% of households lived below the poverty line in Lindi and Mtwara regions [27]. The HIV prevalence rates (categorized) for adults age 15–49 years in Lindi and Mtwara regions were estimated to be 4–6% and 7–10% respectively in 2003/4 [27].

The public health system comprises a network of dispensaries, health centres and hospitals offering a varying quality of care [15]. Nearly all (99%) pregnant women attend antenatal care at least once, and around half of women deliver with a skilled attendant [14].

Between June and October 2007, a survey team of over 200 field staff visited all 243,612 households in the five study districts. Household heads were asked to give their written consent to participate. In a few households (15,823, 7%), nobody could be found on the day of the survey despite repeat visits by interviewers within the day. Over 99% (225,980) agreed to take part. Female participants age 13–49 who had had a live birth in the year before the survey were then separately asked for written consent to participate and were asked questions relating to use of antenatal care, intrapartum care (such as place of childbirth and birth attendant) and postpartum care including essential newborn care indicators, for the most recent birth. Some questions were only asked to women who had had a non-facility delivery.

The questionnaire was administered in Swahili using handheld computers (personal digital assistants or PDA) to capture responses [28]. Standard range, consistency and completeness checks were carried out in the field. Analysis was conducted in Stata version 10 [29].
